# Single‐Cell Transcriptomic Atlas Reveals Metabolic Reprogramming and Transdifferentiation Trajectories in Tubular Epithelial Cells During Chronic Kidney Disease Progression

**DOI:** 10.1155/ijog/9747425

**Published:** 2026-04-17

**Authors:** Xinhui Mao, Minggang Wei, Yilin Li, Ping Xia

**Affiliations:** ^1^ Nephrology Department, Baoying County People’s Hospital, No.1 Xincheng Road Baoying County, Yangzhou, Jiangsu, 225800, China; ^2^ Department of Traditional Chinese Medicine, The First Affiliated Hospital of Soochow University, No.899 Pinghai Road Gusu District, Suzhou, Jiangsu, 215006, China, sdfyy.cn; ^3^ Department of Nephrology, Suzhou Traditional Chinese Medicine Hospital Affiliated to Nanjing University of Chinese Medicine, Suzhou, Jiangsu, China

**Keywords:** metabolic reprogramming, proximal tubule heterogeneity, single-cell RNA sequencing, chronic kidney disease, tubular epithelial cells

## Abstract

**Background:**

In chronic kidney disease (CKD), there is evidence of loss of function and fibrosis in the progression of tubular epithelial cells; however, the cellular heterogeneity and underlying molecular mechanisms are not well defined. Knowledge of the diversity among tubular cells is essential for precision medicine therapy.

**Methods:**

We subjected renal cells from CKD patients to single‐cell RNA sequencing with a focus on tubular epithelial populations. Through unsupervised clustering, a computational pipeline that includes metabolic pathway scoring and pseudotemporal trajectory inference combined with machine learning classification, our analysis enabled the characterization of intratumoral cellular diversity and metabolic states coupled to differentiation trajectories. We analyzed segment‐specific markers (SLC34A1, SLC5A2, LRP2, CUBN, ALDOB, and GATM) and metabolic enzymes (glycolysis and TCA cycle enzymes) in cell subsets.

**Results:**

Tubular cells showed marked heterogeneity and metabolic reprogramming from oxidative phosphorylation to glycolysis, clustering as OXPHOS‐high, glycolytic, dormant, and intermediate cell states. The expression of segment‐specific markers was differentially retained and lost, reflecting maintenance of fate as well as dedifferentiation. Pseudotemporal analysis demonstrated progressive cellular transitions driven by the expression of critical genes such as MALAT1 and ANXA1. Proximal tubule cells constituted ∼70% of the profiled cells with different transcriptomic signatures. Moderate classification results were obtained using a machine learning approach (ROC AUC = 0.673).

**Conclusions:**

This work furnishes a molecular atlas of tubular epithelial cell heterogeneity in CKD, highlighting metabolic reprogramming and transdifferentiation as key processes driving tubular dysfunction and fibrosis. These results also revealed potential therapeutic targets to retain tubular function and ameliorate the progression of CKD.

## 1. Introduction

Chronic kidney disease (CKD) is a predominant worldwide public health problem with an estimated prevalence that affects nearly 10% of the global population [1, 2] and is characterized by a gradual decline in renal function to end‐stage kidney disease requiring dialysis or transplantation. The in vivo pathophysiology of CKD involves a complex cascade of cellular and molecular responses, including tubular epithelial cell injury inflammation, fibroblast activation, and eventual progressive interstitial fibrosis. In these processes, the tubular epithelial cell dysfunction takes a central and frequently initiating part in disease progression because the cells have essential renal functions, such as regulation of fluid and electrolyte balance, reabsorption of metabolites, and disposal of waste.

The renal tubular system has exceptional structural and functional complexity, comprising, while it is unthinkable with the various segments (proximal tubule, loop of Henle, distal convoluted tubule, and collecting duct), of specialized physiological roles. The proximal tubule is especially metabolically demanding and susceptible to injury because of its extensive reabsorptory processes and high ATP demands [3, 4]. Tubular epithelial cells experience dramatic phenotypic changes such as dedifferentiation, loss of differentiation markers, cell cycle arrest, metabolic disturbance, and possibly transdifferentiation into fibroblast‐like cells, including EMT in the progression of CKD [5, 6]. However, the cellular heterogeneity of diseased tubules and the molecular drivers that mediate these pathological transitions are incompletely understood.

Conventional bulk tissue analysis methods have greatly contributed to our understanding of CKD pathophysiology but are hampered by an inability to resolve cell heterogeneity and recognize discrete cell states in complex tissue environments. Over the last years, the emergence of single‐cell RNA sequencing (scRNA‐seq) has radically changed our capacity to dissect cellular diversity at unparalleled resolution in terms of characterization of rare cell populations, exploration of differentiation trajectories, or mapping of communication networks between cells [7, 8]. The application of scRNA‐seq to kidney research in recent years has just begun to uncover the cellular heterogeneity of healthy and diseased kidneys, including previously unrecognized cell types, transitional states, and disease‐associated cellular phenotypes [9, 10].

Metabolic reprogramming is now established as an important aspect of tubular epithelial cell dysfunction in CKD [11, 12]. Healthy proximal tubule cells are mostly dependent on oxidative phosphorylation (OXPHOS) driven by fatty acid oxidation to fuel their high demand for energy related to the active transport processes [3, 13]. Metabolic reprogramming toward glycolysis also occurs as an adaptation in tubular cells during injury and progression of renal disease, similar to the Warburg effect seen in cancer [14]. This metabolic reprogramming is presumed to be both an adaptive response to cellular stress and a maladaptive contributing factor in cell dysfunction and fibrotic stiffening. Nevertheless, how much such metabolism heterogeneity exists when applied to a tubular cell population and its relationship with functional states and the progression of diseases are scarcely known.

Here, we utilized single‐cell transcriptomic profiling to systematically dissect tubular epithelial cell heterogeneity in CKD kidneys. By employing integrated computational methodologies, such as unsupervised clustering, reference‐based annotation, metabolic pathway analysis, pseudotemporal trajectory inference, and machine learning classification we sought to (1) delineate the cell landscape and define distinct tubular cell subpopulations within diseased kidneys, (2) portray expression profiles of segment‐specific markers and determine preservation or ablation of tubular identity, (3) quantitate metabolic heterogeneity map metabolic states across cellular compartments, (4) reconstruct differentiation and transdifferentiation trajectories occurring during CKD progression, and (5) identify cardinal molecular drivers associated with signaling networks underlying tubular dysfunction. Our data define a molecular high‐resolution atlas of tubular epithelial cell diversity in CKD and highlight metabolic reprogramming and cellular transdifferentiation as central disease pathogenic mechanisms, providing opportunities for the development of targeted therapies.

## 2. Methods

### 2.1. Sample Collection and Preparation

We harvested kidney tissue from CKD patients and subjected it to scRNA‐seq. Fresh tissue samples were immediately dissociated into single cell suspensions enzymatically using digestion protocols optimized for kidney tissue. Disaggregated cells were then filtered using proper sieves to get rid of debris and clusters, allowing for good single cell suspensions. Cell viability was determined by trypan blue exclusion, and only those samples whose viability exceeded 80% were analyzed in sequencing experiments.

### 2.2. scRNA‐seq

scRNA‐seq libraries were generated following the established procedures. Single cells were isolated and barcoded, reverse‐transcribed, and amplified on cDNA. Libraries for sequencing were prepared and sequenced on platforms with high‐throughput capability to obtain enough depth in sequencing for full‐scale transcriptomic profiling. Raw reads of sequencing were demultiplexed and aligned to the human reference genome for quantification of gene expression matrices. A rigorous quality control process was carried out to insure the accuracy of the data. Cells were filtered based on several factors, such as the number of detected genes, nFeature_RNA (nFeature RNA), total RNA counts, nCount RNA, and mitochondrial gene percentage (percent. mt). Cells exhibiting particularly low or high feature counts, suggestive of being doublets or low‐quality cells, were removed from the analysis. Additionally, cells with mitochondrial transcript proportions exceeding predetermined thresholds were filtered to remove damaged or stressed cells. Ribosomal gene percentages (percent.ribo) were also calculated and evaluated as part of quality assessment. Gene expression matrices were normalized to account for sequencing depth variations across cells. Principal component analysis (PCA) was performed to reduce the dimensionality of the gene expression data. The optimal number of principal components was determined using elbow plots, which visualize the relationship between principal components and explained variance. Uniform Manifold Approximation and Projection (UMAP) was subsequently applied for two‐dimensional visualization of cellular heterogeneity. Unsupervised clustering was performed using graph‐based clustering algorithms, with resolution parameters optimized to balance cluster granularity and biological interpretability. Multiple clustering resolutions were tested, with resolution 0.8 selected for the primary analysis based on cluster stability and biological coherence.

### 2.3. Cell Type Identification and Annotation

Cell types were identified by a combination of methods. Unsupervised clustering results were first manually annotated according to their expression of known cell type‐specific marker genes. Cell types were assigned by reference‐based prediction to annotated mouse kidney cell atlases [15–17] to obtain automatic cell type predictions. Prominent types of renal cells were delineated such as proximal tubule epithelial cells, distal convoluted tubule cells, collecting ducts, endothelial cells, fibroblasts pericytes podocytes, and immune cell including macrophages and T cells. We used interpretable biological names (OXPHOS‐high PT, glycolytic PT, and injury‐associated PT) for annotated cell clusters rather than numerical cluster identities. Segment‐specific proximal tubule zoning markers such as SLC34A1, SLC5A2, LRP2 (megalin), CUBN, ALDOB, and GATM were tested to detect heterogeneity and segmental identity of PT.

### 2.4. Differential Gene Expression Analysis

Differential gene expression analysis was conducted to identify genes that distinguish cellular clusters and functional states. For each cluster comparison, statistical tests were performed to identify significantly upregulated or downregulated genes. Gene expression patterns were visualized using various approaches including UMAP feature plots, violin plots, dot plots, and heatmaps. Hierarchical clustering was applied to gene expression matrices to identify coordinated gene modules and functional signatures across cellular subpopulations.

### 2.5. Metabolic Pathway Analysis

Scoring was used to evaluate glycolysis and OXPHOS activity for individual cells. Pathway‐based enrichment term used was based on custom‐made gene sets (in comparison, classical databases were mainly curated) and computed as the sum of expression values for genes associated with the same pathway. Cells were assigned to OXPHOS‐high, glycolytic, dormant/mitochondrial‐high, and intermediate states according to their pathway scores. Furthermore, the expression of glycolytic enzymes (GPI, ENO1, HK1, LDHA, and LDHB) and the TCA cycle enzyme (IDH1 and IDH2), as individual metabolic enzymes, was evaluated to determine molecular heterogeneity in metabolism.

### 2.6. Pseudotime Trajectory Analysis

Pseudotemporal ordering of cells was performed to infer differentiation trajectories and disease progression dynamics. Trajectory inference algorithms were applied to construct continuous paths through the cellular state space, with cells ordered along pseudotime representing their position along developmental or pathological transitions. Stretched pseudotime distributions were generated for each cluster to visualize temporal heterogeneity and identify early‐stage versus late‐stage cellular states. Gene dynamics along pseudotime trajectories were analyzed to identify driver genes and transcriptional programs associated with cellular transitions.

### 2.7. Functional Landscape Analysis

Functional landscape analysis was used to identify genes and pathways that are important (variable) and conserved between cellular states. Scores of known genes on gene variability across clusters or pseudotime trajectory as well as consistency among individual cell types were defined. Importance versus conservation scatter plots were used to rank biological processes and regulatory genes that underlie cellular heterogeneity and disease progression.

### 2.8. Cell–Cell Communication Analysis

Intercellular communication networks were characterized by ligand–receptor interaction analysis. Known ligand–receptor pairs were explored systematically for different sender and receiver cell type combinations with levels of expression and probability of interaction being estimated on the basis of coexpression between ligands (sender) and receptors (receiver). Interaction profiles were displayed in dot plots, with the size of dots representing fraction of cells expressing the interaction pair and color depth showing average expression levels.

### 2.9. Machine Learning Classification

Automated cell type classification was achieved using machine learning models. Cell type classification was performed using a Random Forest classifier implemented in the R package randomForest (v4.7‐1.1), with 500 trees and the default mtry setting. The top 2000 variable genes as calculated by variance‐stabilizing transformation were used for feature selection. Training datasets were created from cells that had been manually annotated, and gene expression profile‐based features were derived. Classification algorithms were built and tested using fivefold cross‐validation methods. Model performance was assessed with receiver operating characteristic (ROC) and precision–recall curves, and the area under the curve (AUC) measure was used to discriminate. Learning curves were used to measure convergence to test whether more training data would improve the model. The learned classifiers were then used to identify cellular identities on the overall dataset.

### 2.10. Statistical Analysis and Visualization

All statistical analyses were conducted using the appropriate software and programming. Relationships between quality control metrics and biological attributes were analyzed by correlation analysis. We conducted proportional composition analysis to calculate proportions of cell types and different metabolic states in each sample or condition. The outcomes were presented by several kinds of plots, such as UMAP, violin, density, scatter dot bar, pie, stacked bar, heatmap, and dot charts. All visualizations were created with uniform color scales and easily understandable annotations.

## 3. Results

### 3.1. Single‐Cell Transcriptomic Profiling Identifies Tubular Epithelial Cell Dissociation and Transdifferentiation in CKD Development

scRNA‐seq analysis of cellular landscape and dynamic changes in infiltrating renal tubular epithelial cells during experimental CKD. Quality control and dimension reduction were performed to optimize the clustering parameters of single cells, resulting in clusters with obvious partitioning characteristics in low‐dimensional space. Several complementary analytical methods, including unsupervised clustering and reference‐based cell type prediction, revealed major renal cell populations such as the tubular epithelial cells, fibroblasts, endothelial cells, pericytes, and immune cells. Deeper characterization revealed extensive heterogeneity within the tubular compartment, with cells covering many functional and transition states. Trajectory analysis found hypothetical transdifferentiation pathways, which may indicate a stepwise transition between the phenotypes of disease progression. Metabolic profiling further revealed that distinct cellular subpopulations possessed specific metabolic profiles, including OXPHOS‐like, glycolytic‐like, and even dormant or intermediate metabolic phenotypes. These results are of particular importance in understanding the cellular and molecular pathomechanism of tubular dysfunction and fibrotic remodeling in CKD (Figure 1).

FIGURE 1Single‐cell transcriptome analysis to identify cellular heterogeneity and metabolic reprogramming of CKD kidneys. (a) Elbow plot illustrating the relationship between principal components and variance for estimating optimal dimensionality for downstream analysis. (b) UMAP visualization of all the cells colored based on unsupervised clustering (resolution = 0.8), showing clear cell populations. (c) Expanded UMAP plot of the same cells with cluster annotation. (d) Reference‐based prediction cell type annotation of major renal cell types: tubular cells (CAF and ICC), endothelial cells (Endo), fibroblasts (fib), pericytes (peri), macrophages (MΦ), and T cells (TC). (e) UMAP projection colored by cell state or quantile of trajectory stage (numbered 1–5), associated with potential transdifferentiation routes. (f) Annotation of metabolic state illustrating different metabolic phenotypes, such as OXPHOS‐high, glycolytic (Glyc), dormant/quiescent, and intermediate.

**Figure:**
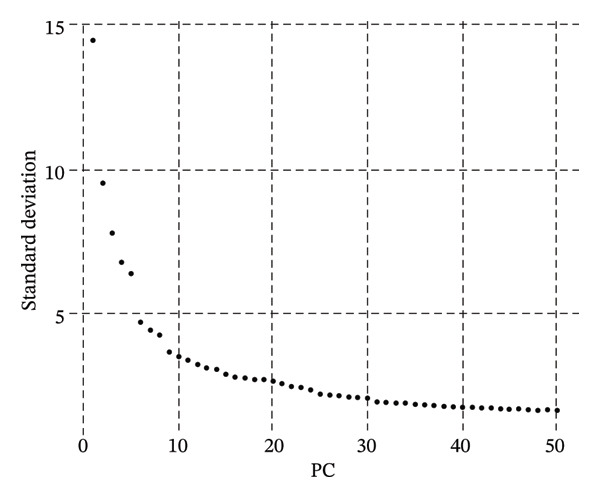
(a)

**Figure:**
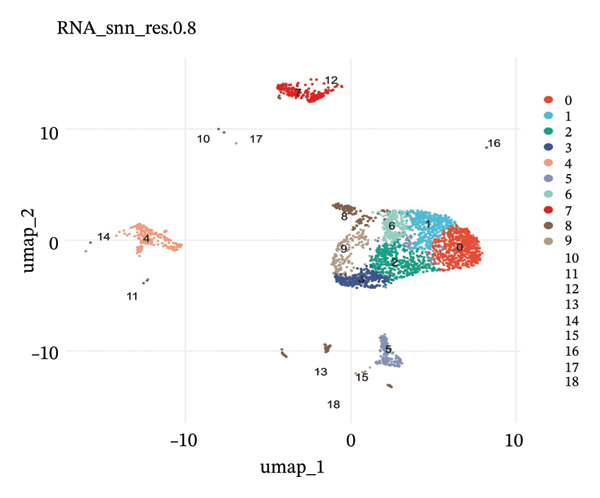
(b)

**Figure:**
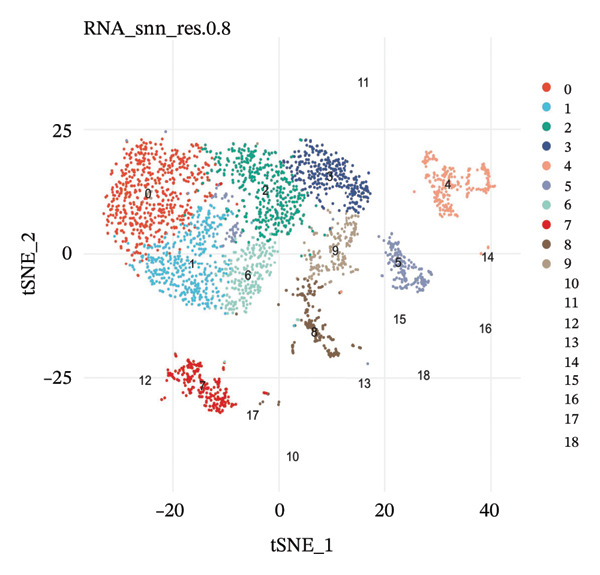
(c)

**Figure:**
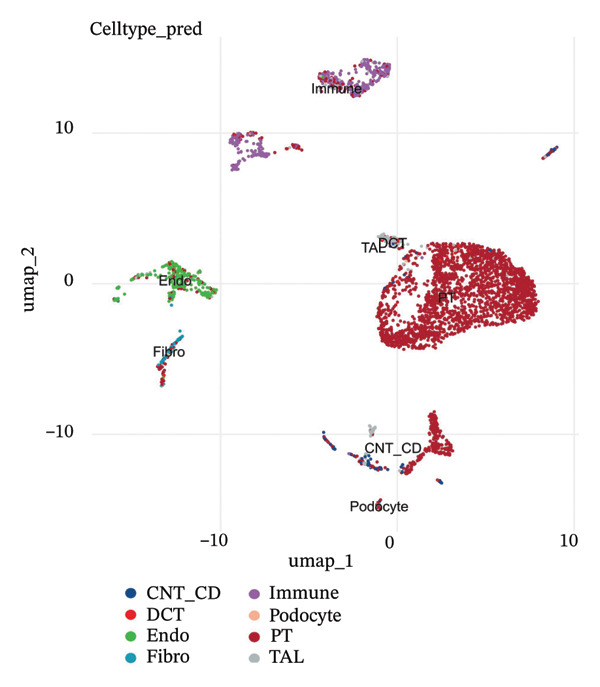
(d)

**Figure:**
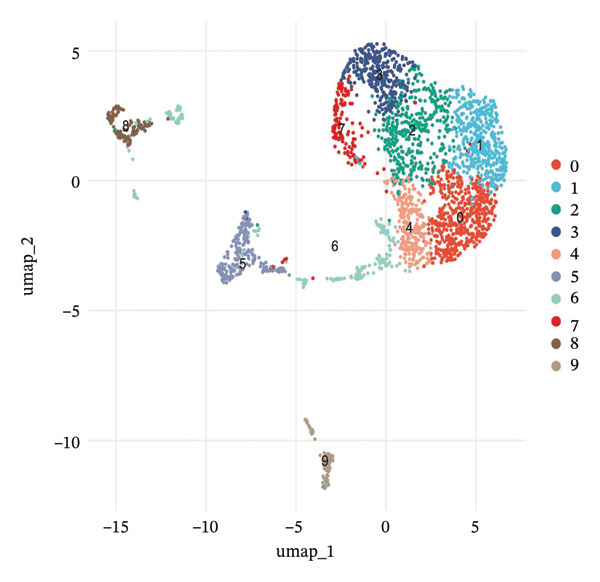
(e)

**Figure:**
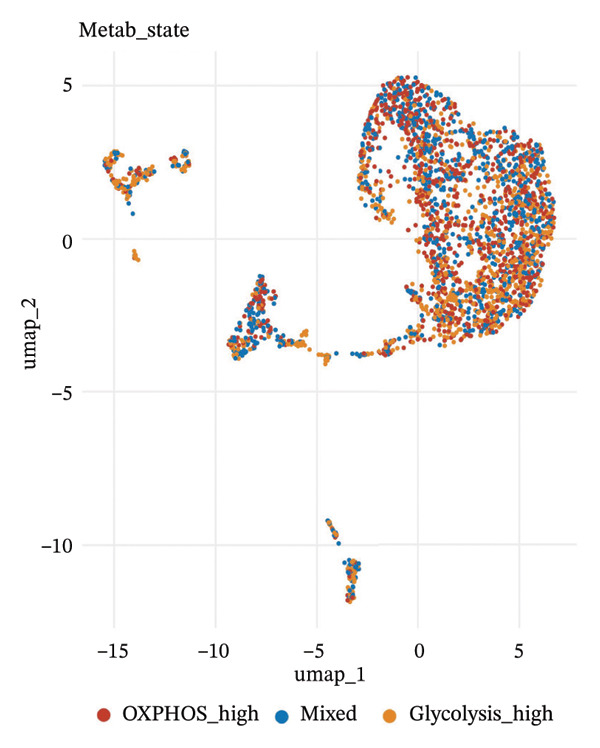
(f)

### 3.2. Molecular Definition of Proximal Tubule Heterogeneity and Segmental Identity in CKD

To delineate heterogeneity within tubular epithelial cells, the expression of major segment marker genes was plotted over the single cell landscape. The observed heterogeneous zonation of proximal tubule markers reflects rather the unique functions need *d* to accomplish along the PT. Another group of Na(+) transporters, SLC34A1 (Na‐Pi cotransporter) and SLC5A2 (Na‐glucose cotransporter), highly expressed in the early proximal tubule (S1 segment), was selectively enriched in certain cellular subpopulations, suggesting the retention of segmental identity in a portion of tubular cells. Other PT markers, such as LRP2 (megalin) and CUBN (cubilin), involved in the reabsorption of proteins were more widely expressed across PT cells with different degrees of intensity, perhaps indicating a functional heterogeneity even within the same segment. ALDOB, a fructose‐metabolizing enzyme and known specific marker of the proximal tubule that is expressed in a region‐specific manner (79), as well as GATM, involved in creatine synthesis, had also relatively specific patterns of expression that further distinguished subpopulations of PVs. Importantly, the spatial distribution of these markers served to highlight that while some cells had retained robust expression of a number of proximal tubule markers associated with healthy differentiated phenotypes, others demonstrated diminished or complete loss of such gene expression, indicative of an attenuated maintenance in segment‐specific identity as disease developed. This molecular heterogeneity also reflects the diverse cellular responses and possible dedifferentiation or transdifferentiation events that take place in tubular epithelial cells when CKD develops (Figure 2).

FIGURE 2Proximal tubule segment‐specific markers show cellular heterogeneity in the CKD kidneys. UMAP representations of the expression profile of key proximal tubule markers. (a) SLC34A1 (sodium phosphate cotransporter) expression. (b) Expression of SLC5A2 (sodium glucose cotransporter 2), identifying early proximal tubule (S1 segment). (c) LRP2 (megalin), responsible for protein reabsorption. (d) Expression of CUBN (cubilin), a critical subunit in the megalin–cubilin receptor complex. (e) ALDOB (aldolase B) is related to fructose metabolism. (f) Expression of GATM (glycine amidinotransferase) in creatine biosynthesis. The degree of color represents the relative expression levels of that gene (darker blue/purple = higher) after normalization. The heterogeneity corresponds to segmental identity and functional diversity among proximal tubule cells and with a putative loss of differentiation markers during the course of disease.

**Figure:**
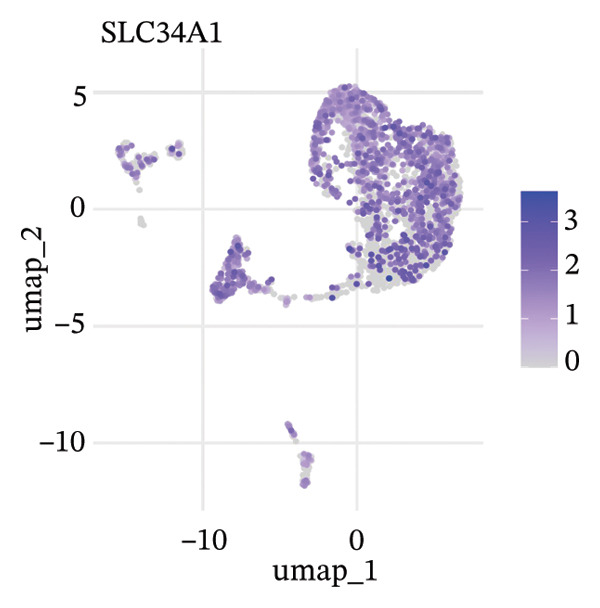
(a)

**Figure:**
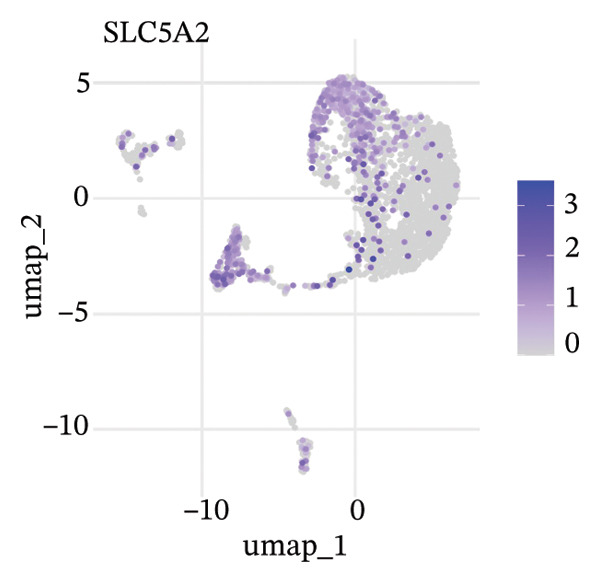
(b)

**Figure:**
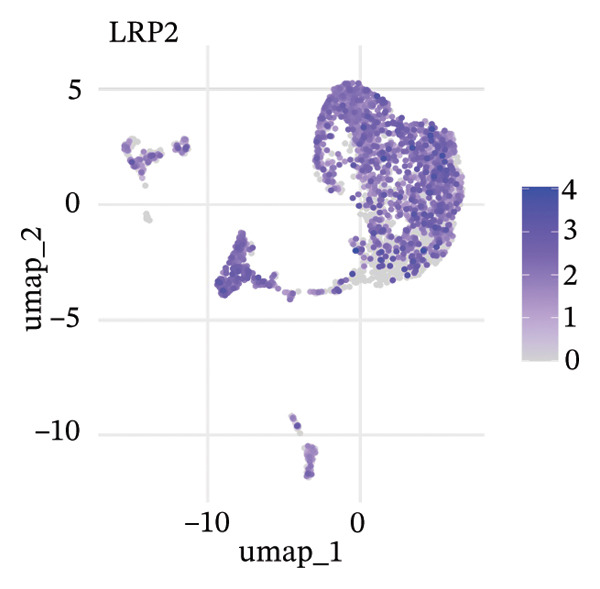
(c)

**Figure:**
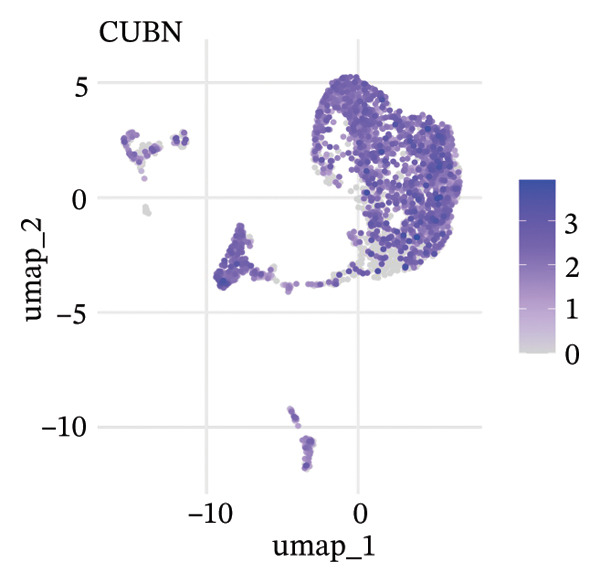
(d)

**Figure:**
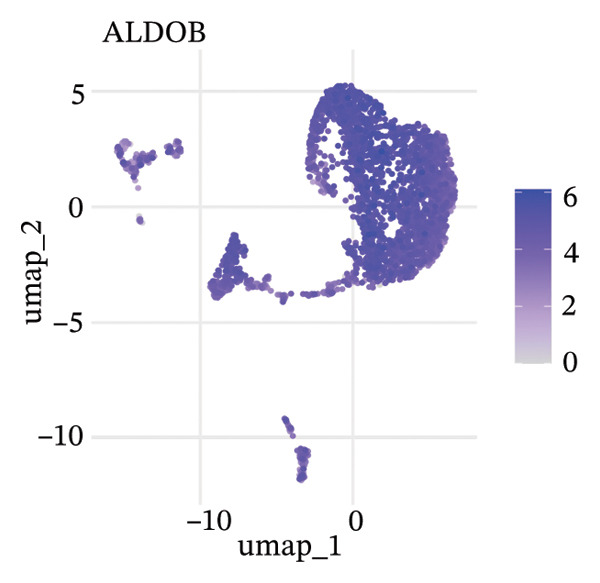
(e)

**Figure:**
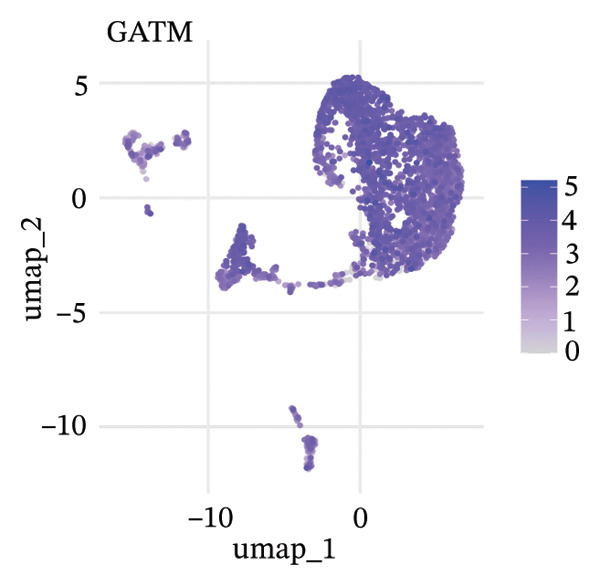
(f)

### 3.3. Metabolic Remodeling and Cellular Crosstalk Drive Epithelial Tubules in CKD

We conducted unsupervised comprehensive metabolic pathway scoring and cell–cell interaction analysis of the underlying changes driving tubular cell heterogeneity with CKD development. Scores of the glycolysis and OXPHOS pathways disclosed a clear metaphenetic separation among tubular cells, in which specific subclusters displayed glycolytic‐ or OXPHOS‐dominant phenotypes than did some between‐state clusters. This metabolic conversion implies that in the progression of disease, tubular epithelial cells adaptively reprogram metabolism from OXPHOS to glycolysis, which is attributed to cellular stress responses and fibrotic transition. The analysis of ligand–receptor interaction between different cell type proportions showed the central signaling pathways regulating intercellular communications, the expression patterns of which were dependent on cellular contexts as well as diseases. The quantitative estimation of the composition of cell types showed that most of the cells in the dataset were assigned to clear metabolic and functional states, proximal tubule‐like being the predominant one. When annotated by the metabolic state, glycolytic cells comprised a significant fraction, consistent with the metabolic shift observed over CKD development. A comparison of differentially expressed genes in proximal tubule subpopulations highlighted unique transcriptional signatures with correlated upregulation or downregulation of gene modules. Importantly, the proportion comparison between PT clusters illustrated a gradient change of cell composition: while some clusters became dominated by injury‐associated and profibrotic signatures, others remained more differentiated. Taken together, our results illustrate that metabolic reprogramming and derangement cell–cell communication networks represent the main causes for tubular epithelial cell heterogeneity and maladaptive responses in CKD (Figures 3(a), 3(b), 3(c), 3(d), and 3(e), 3(f)).

FIGURE 3Metabolic reprogramming and cell composition analysis show different functional states of CKD tubular cells. (a) Scatter plot of cells based on glycolysis versus OXPHOS (oxidative phosphorylation) pathway scores. The cells are color coded according to their metabolic classification: red representing glycolysis‐dominant and green OXPHOS‐dominant cells. (b) Dot plot to visualize ligand–receptor interaction patterns between different cell types (proportions). Dot size represents the proportion of cells with the interaction pair, and color intensity shows mean expression. (c) Bar plot representing the percentage of cells in each category, with dominant groups being depicted in bright green. (d) Pie chart showing the relative cell type distribution, with proportion of PT (proximal tubule)‐related cells in yellow–green and other cell types colored differently. (e) Heatmap showing the differential expression of genes across cells with rows corresponding to genes and columns to cells (cluster ordered). Blue represents low expression, and orange/red indicates high expression. (f) The percent makeup of PT clusters across samples or conditions represented as a stacked bar chart (in colors indicating different metabolic or injury states: Inj, red; OXPHOS, blue; and Glyc/Mt_Pt, green).

**Figure:**
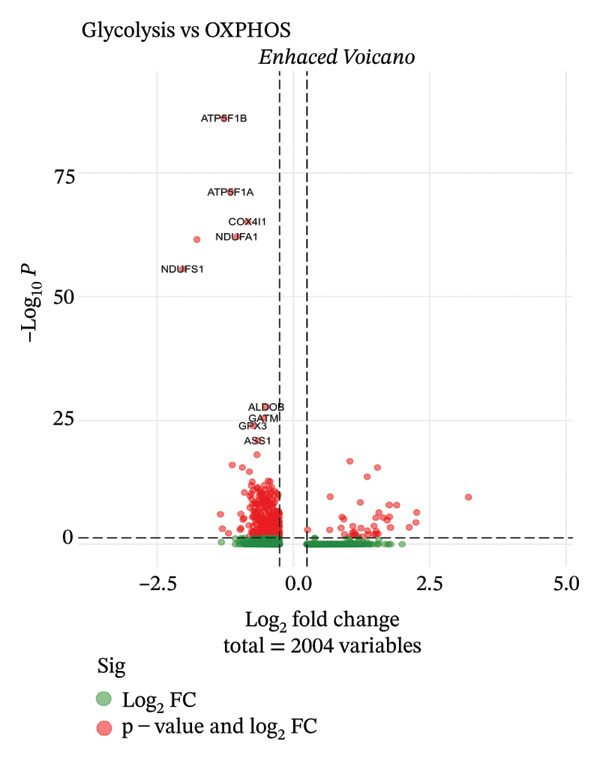
(a)

**Figure:**
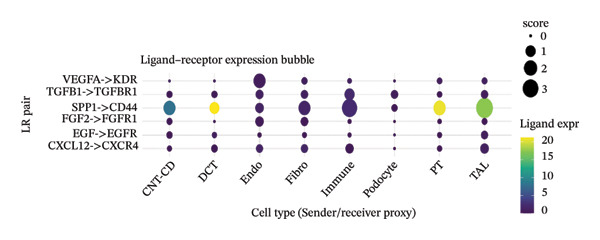
(b)

**Figure:**
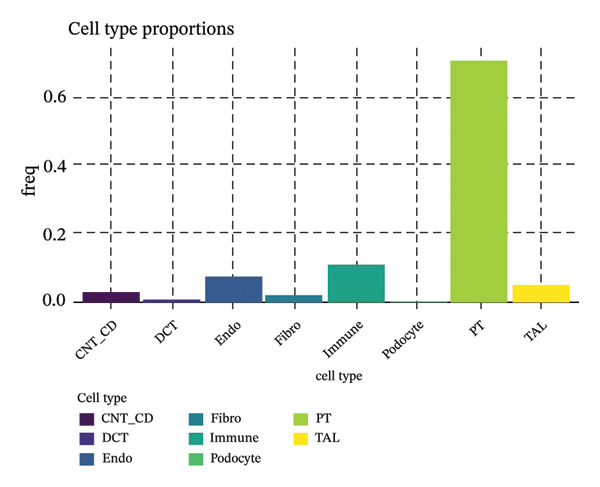
(c)

**Figure:**
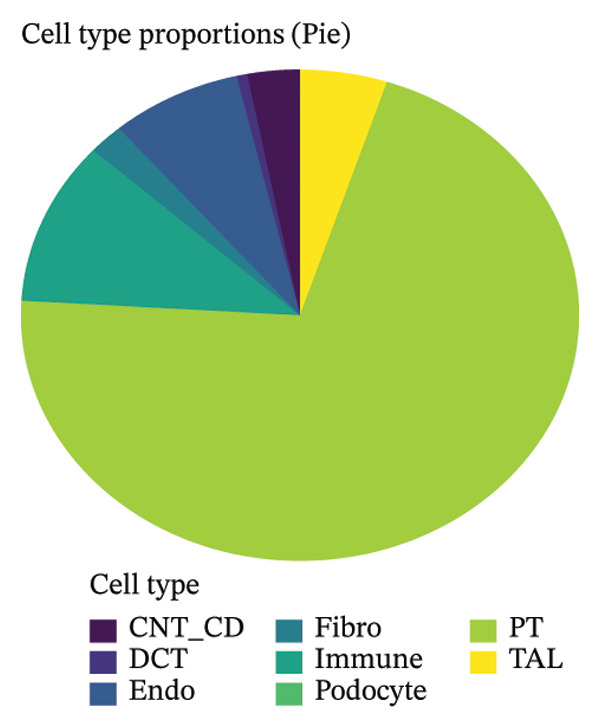
(d)

**Figure:**
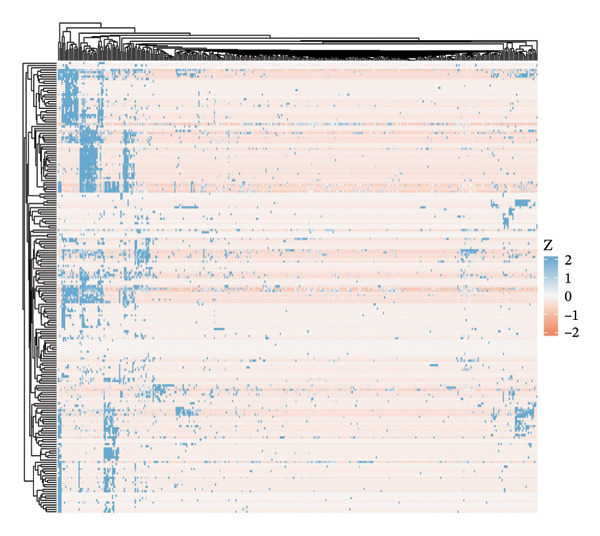
(e)

**Figure:**
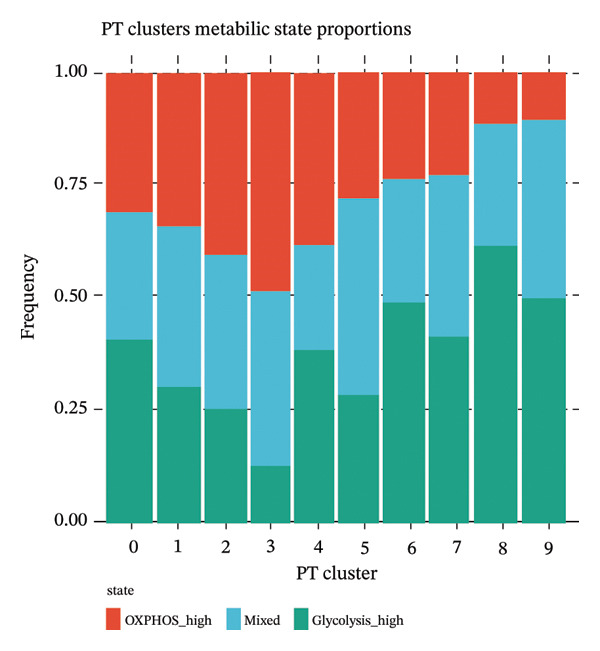
(f)

### 3.4. QC and Metabolic Signatures Characterize Tubular Cell States in CKD

Extensive quality control analysis described the technical and biological attributes of the single‐cell dataset. The gene detection statistics (nFeature_RNA) and total RNA counts (nCount_RNA) were displayed by cell type and demonstrated the difference in transcriptional activity and metabolic status among these cells. The analysis of the mitochondrial gene percentage showed significant heterogeneity, and in some populations, high levels of mitochondrial transcripts were observed—an indicator for cellular stress and metabolic derangement in CKD‐affected tubular epithelial cells. Variations in HK1 (hexokinase 1) expression among cellular identities argued for metabolic heterogeneity and supported the notion that metabolic reprogramming is a key element of CKD progression (Figures 4(a), 4(b), 4(c), 4(d), 4(e), and 4(f)).

FIGURE 4QC metrics and metabolic marker expression demonstrate cellular heterogeneity in CKD kidneys. (a)‐(b) Density plots represent cluster distributions for the number of detected genes (nFeature_RNA) and total RNA counts (nCount_RNA), respectively, reflecting differences in transcriptional complexity and metabolic activity. (c) Scatter plot reflecting the correlation between gene features and RNA counts over cell types. (d) The proportion of mitochondrial genes indicates high levels in specific populations, indicative of cellular stress. (e) Dot plot matrices depict cell identity‐specific expression patterns of marker genes. (f) A framework model of heterogeneous glycolytic capabilities in cell subpopulations according to the expression level of HK1. Glycolysis‐ and TCA cycle–related genes.

**Figure:**
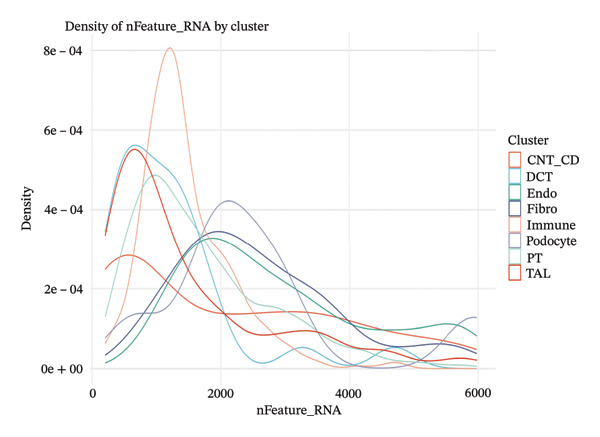
(a)

**Figure:**
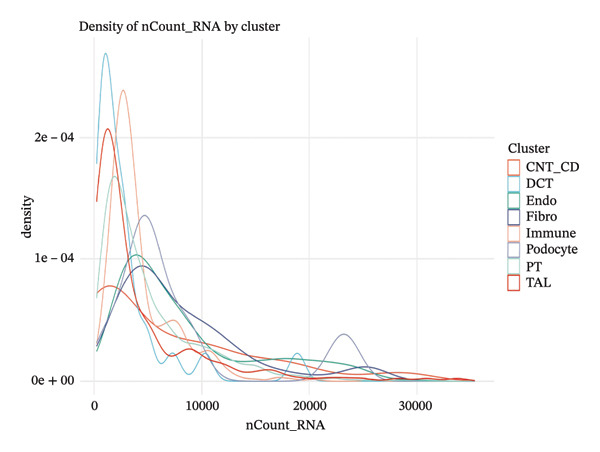
(b)

**Figure:**
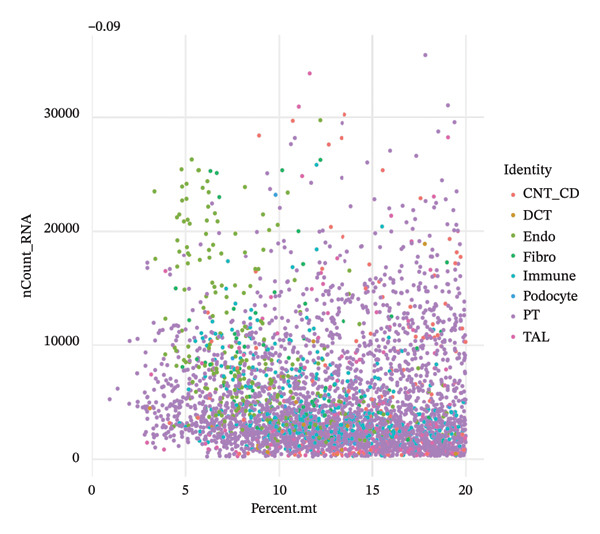
(c)

**Figure:**
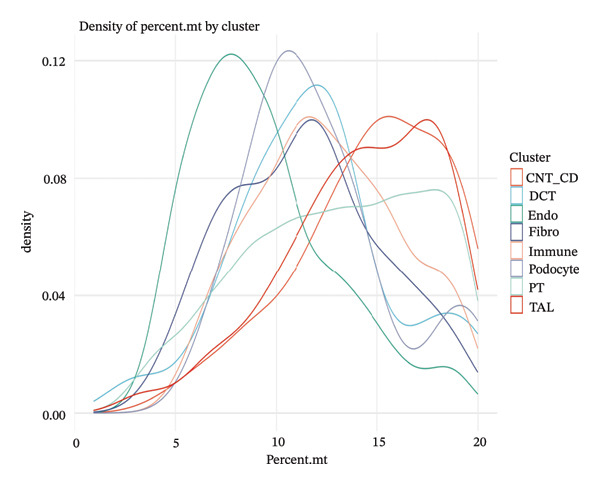
(d)

**Figure:**
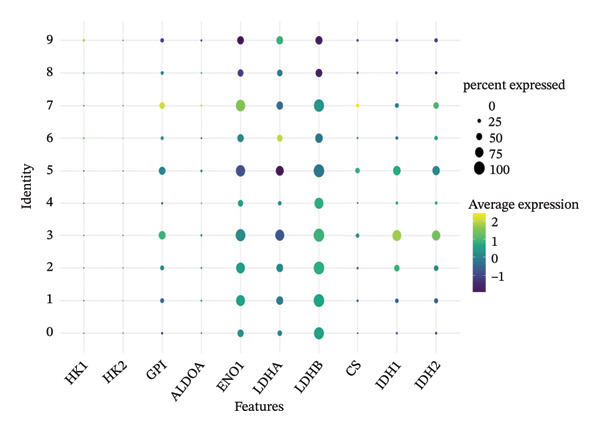
(e)

**Figure:**
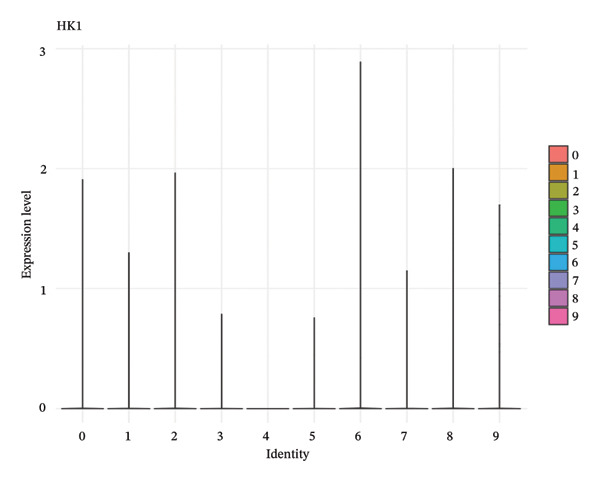
(f)

### 3.5. Enzymes Reveal Metabolic Stratification

Direct measurement of metabolite levels via isotope dilution demonstrated considerable metabolic heterogeneity. The expression of glycolytic enzymes GPI and ENO1 was highly heterogeneous, which possibly reflects different glycolytic potential of tubular cells. Lactate dehydrogenase isoenzymes showed differential expressions: LDHB (pro‐oxidative metabolism) versus LDHA (glycolytic metabolism) suggesting different metabolic strategies. The expression of TCA cycle enzymes IDH1 and IDH2 was also cluster‐specific, with some populations retaining oxidative metabolism while others exhibiting mitochondrial dysfunction. These metabolic enzyme signatures efficiently categorized tubular cells into clearly defined functional states (Figures 5(a), 5(b), 5(c), 5(d), 5(e), and 5(f)).

FIGURE 5Expression of glycolytic and TCA cycle enzymes marked metabolic heterogeneity is apparent across tubular cells, through the expression of both key glycolytic and TCA cycle enzymes. Violin plots representing the expression spread of core metabolic enzymes in Clusters 0–10: (a) GPI (glycolysis second step), (b) ENO1 (penultimate glycolytic step), LDHB (oxidative metabolism), LDHA (glycolytic metabolism), IDH2 (mitochondrial TCA cycle), and IDH1 (cytoplasmic isoform). Expression profiles of individual clusters reflect various metabolic statuses of development during CKD.

**Figure:**
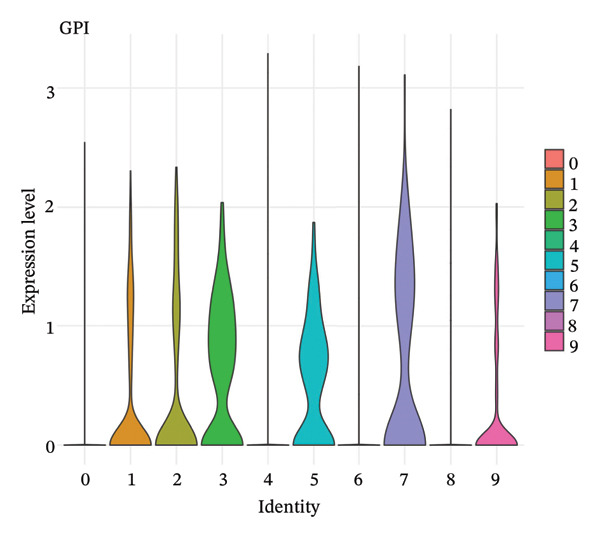
(a)

**Figure:**
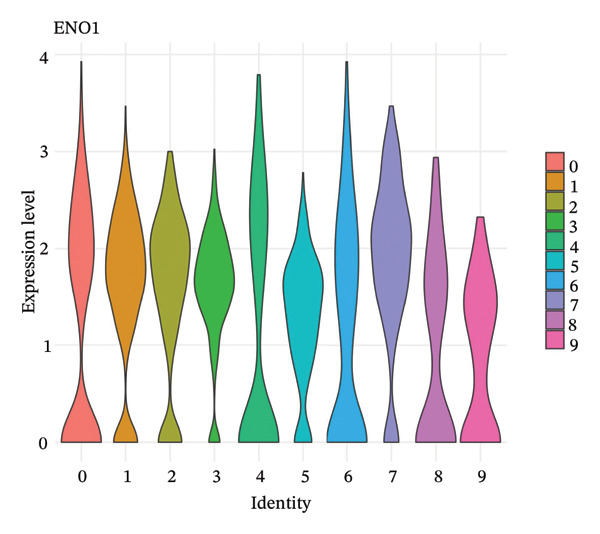
(b)

**Figure:**
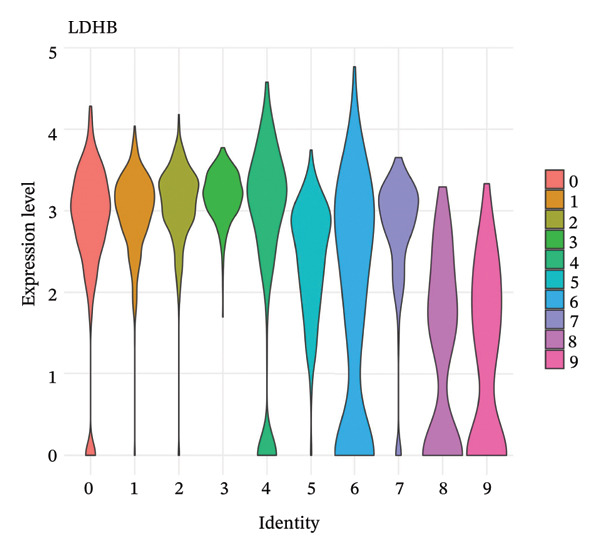
(c)

**Figure:**
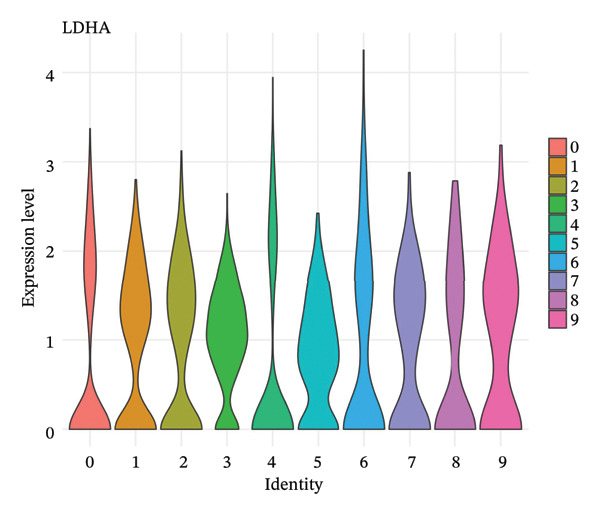
(d)

**Figure:**
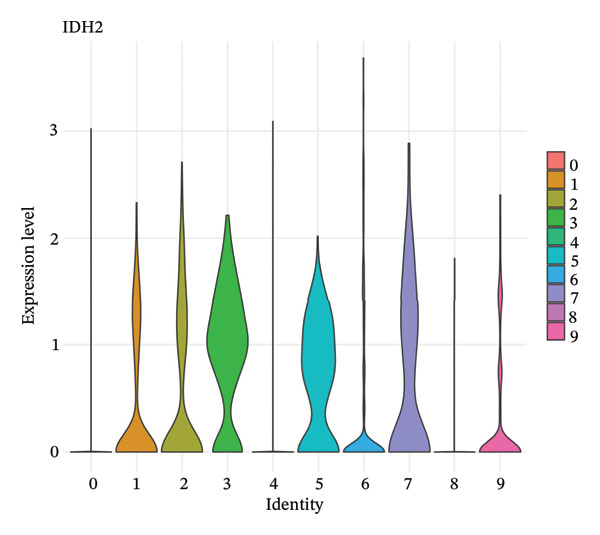
(e)

**Figure:**
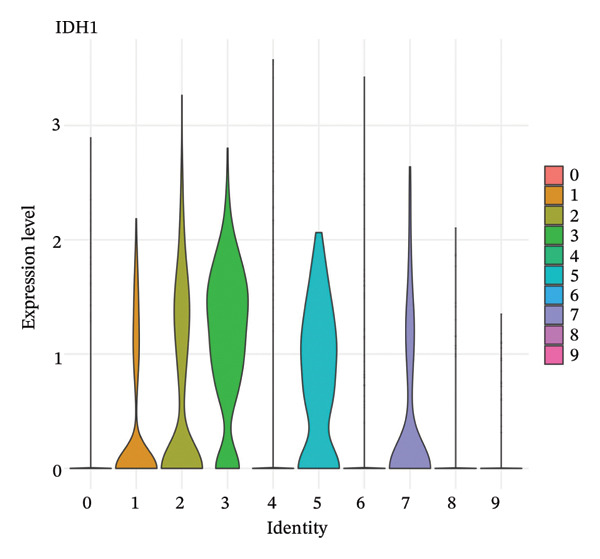
(f)

### 3.6. Proximal Tubule Subpopulations Are Defined by Metabolic State Distribution

Integrated analysis identified significant metabolic heterogeneity among PT subclusters, characterized by different mixtures of OXPHOS‐high, glycolytic, and dormant/mitochondrial‐high cells. Certain clusters preserved an oxidative metabolism, reminiscent of normal cells, while others had glycolytic dominant or mixed patterns indicative of transitional stages. The functional landscape analysis revealed the enriched gene expression modules, which were coordinated with response to injury, metabolic, and transport functions. Cell type‐specific metabolic gene profiling revealed cluster‐dependent signatures for different functional states, indicating that PT cells in CKD can be subgrouped into metabolically programmed subpopulations defined by function (Figures 6(a), 6(b), 6(c), 6(d), 6(e), and 6(f)).

FIGURE 6Metabolic state distribution and functional profiling demonstrate discrete proximal tubule subtypes. (a)‐(b) Stacked bar plots display a proportion of metabolic states across PT clusters: OXPHOS_high (yellow), Glyc (cyan), and dorm/Mt_high (purple). (c) Correlation heatmap showing quality control metric correlation. (d) Hierarchical clustering heatmap of functional landscape and gene expression patterns in PT subclusters. (e) Dot plot matrix visualizes cluster‐pertaining metabolic gene signatures. (f) Through the importance versus conservation metrics, the scatter plot reveals critical biological processes underlying PT heterogeneity.

**Figure:**
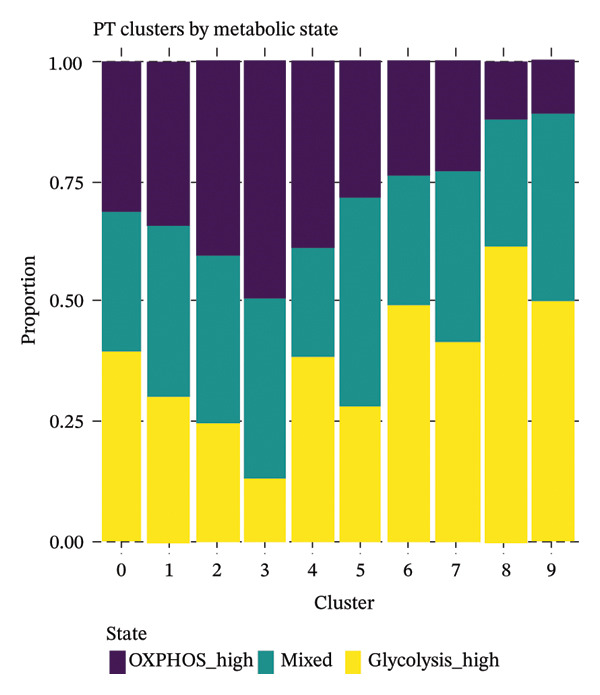
(a)

**Figure:**
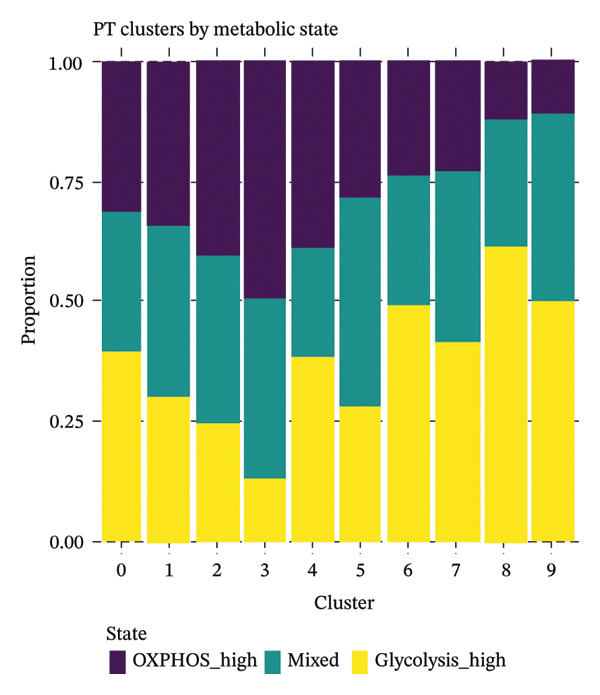
(b)

**Figure:**
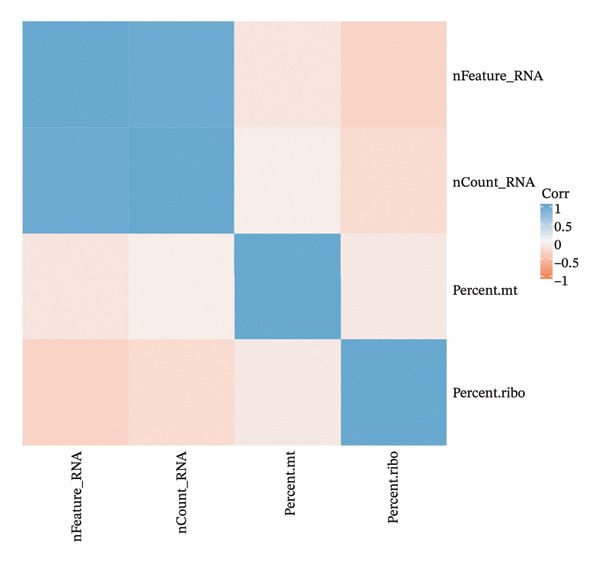
(c)

**Figure:**
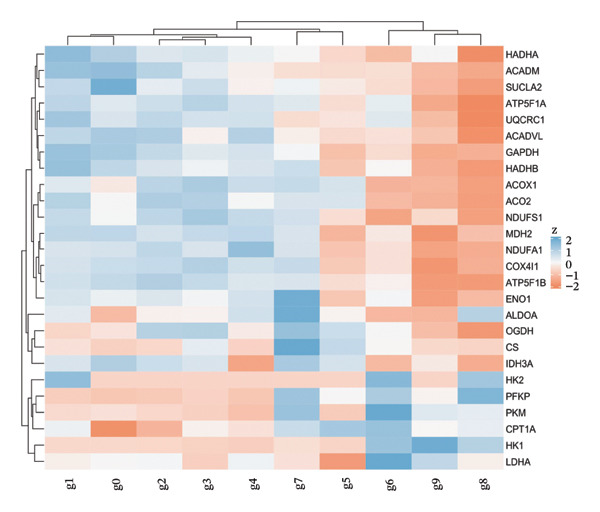
(d)

**Figure:**
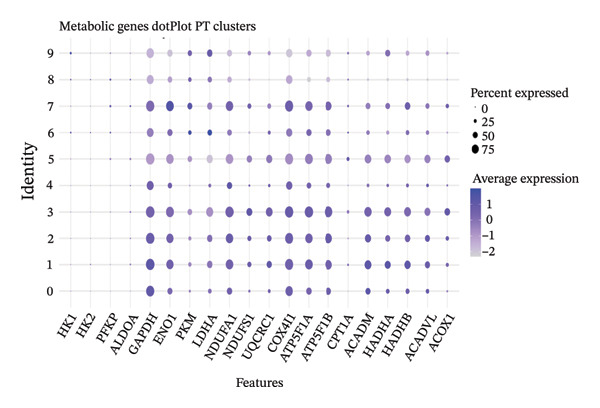
(e)

**Figure:**
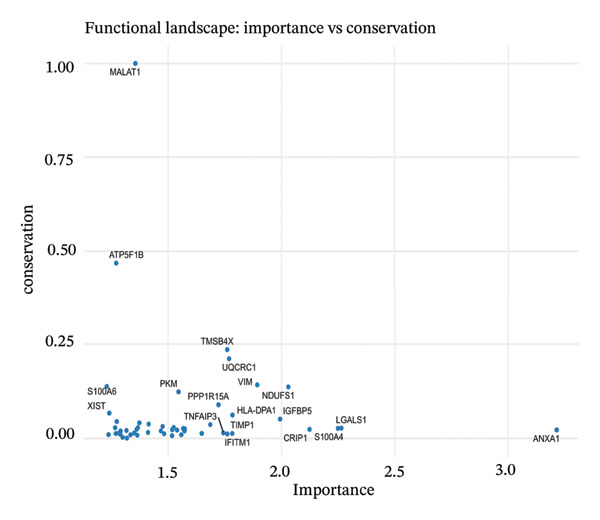
(f)

### 3.7. Pseudotemporal Trajectory Analysis Reveals Progressive Differentiation States

Pseudotime trajectory analysis investigated temporal dynamics of tubular cell states during CKD. Different clusters occupied distinct positions along the disease progression trajectory, with Cluster 0 (OXPHOS‐high PT cells) as the starting point. The temporal ordering suggested continuous phenotypic transitions rather than discrete changes. MALAT1 showed highest conservation across states, while ANXA1 displayed high variability, indicating its role in specific transitions. Machine learning classifiers achieved moderate performance (ROC AUC = 0.673), reflecting the challenge of distinguishing overlapping cell states. Dataset composition showed PT cells as the dominant population (∼70%), consistent with their vulnerability to CKD injury (Figures 7(a), 7(b), 7(c), 7(d), 7(e), and 7(f)).

FIGURE 7Pseudotemporal trajectory and machine learning analysis reveal cellular transitions in CKD kidneys. (a) Stretched pseudotime distribution shows temporal positioning of clusters along disease progression trajectory. (b) Functional landscape plot displays gene importance versus conservation, with MALAT1 showing highest conservation and ANXA1 showing high variability. (c) and (d) ROC and precision–recall curves evaluate classifier performance (ROC AUC = 0.673). (e) Learning curve shows model stability at 0.67 AUC. (f) Bar chart shows PT cells comprising ∼70% of the dataset, followed by smaller populations of other kidney cell types.

**Figure:**
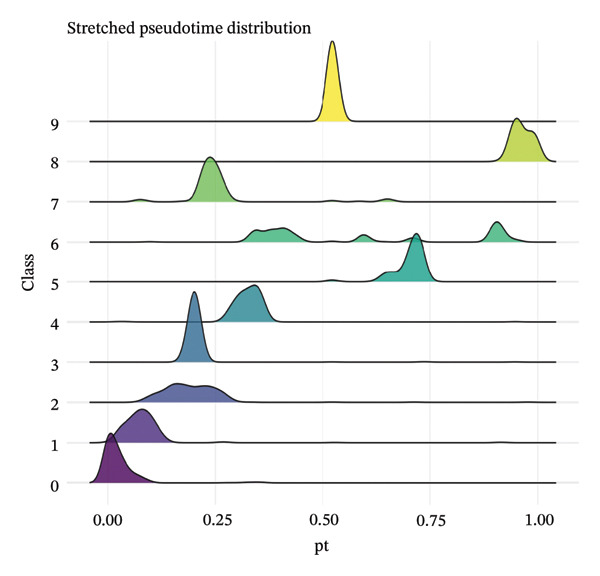
(a)

**Figure:**
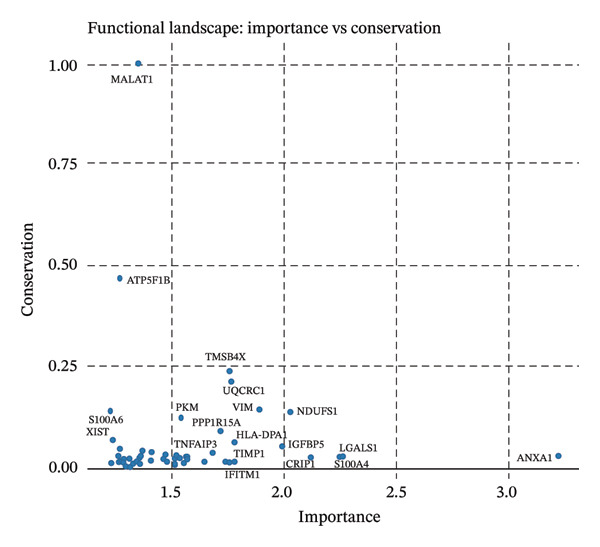
(b)

**Figure:**
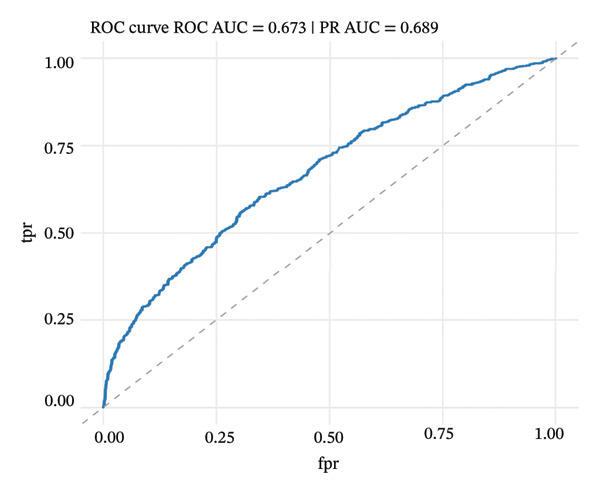
(c)

**Figure:**
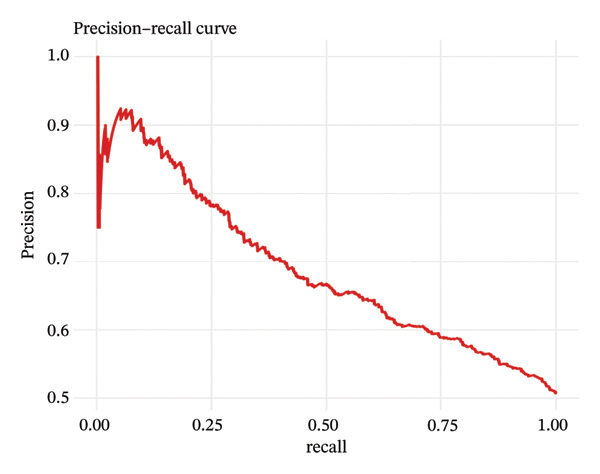
(d)

**Figure:**
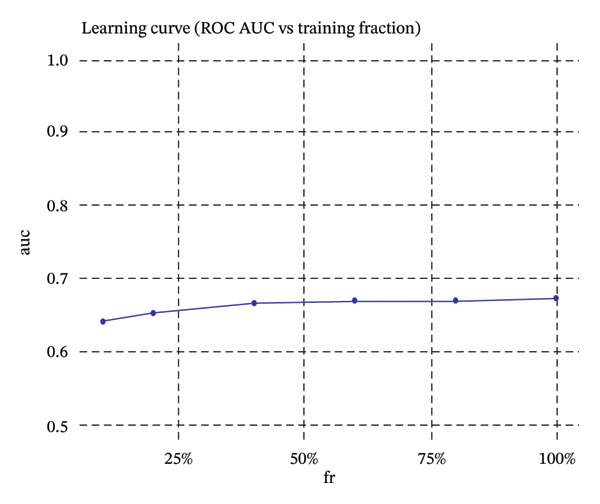
(e)

**Figure:**
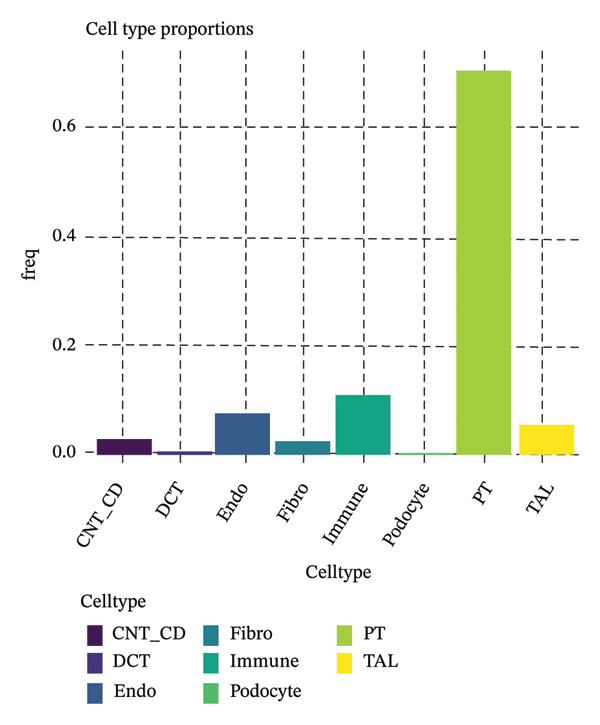
(f)

## 4. Discussion

This work presents an in‐depth single‐cell transcriptomic landscape of tubular epithelial cells in CKD [15, 16] and uncovers previously unanticipated cellular heterogeneity and dynamic state transitions that underpin the hierarchical complexity of the traditional uniformed view of tubular dysfunction. Our results reveal that the disease tubular compartment contains diverse functionally specialized subpopulations, differing in metabolic status, differentiation states, and transcriptional programs. [17]. The identification of continuous cellular transitions along pseudotemporal trajectories, rather than discrete state changes, suggests that tubular dysfunction in CKD is a spectrum of cellular adaptations and maladaptations, rather than on–off healthy versus diseased states. The pronounced heterogeneity within proximal tubule subsets is particularly important since they represent ∼70% of the dataset and are among cells analyzed with a high level of metabolic and transcriptional divergence [18, 19]. This result is consistent with the susceptibility of proximal tubules to injury, such as during ischemia and reperfusion (16), reflecting their high metabolic requirements and intense reabsorptive activity, but it goes further in showing that responses to injury are profoundly heterogenous at the cellular level. Some cells remain highly expressing of segment‐specific markers and oxidative metabolism associated with normal tubular epithelium, whereas others display strong evidence of dedifferentiation and metabolic reprogramming and are poised to transition toward profibrotic phenotypes. One of the most remarkable results in this study is the distinct metabolic segregation that separates the populations of tubular cells OXPHOS‐high, glycolytic, dormant‐mitochondrial high, and intermediary. This metabolic diversity is consistent with and extends previous bulk tissue studies showing that tubular cells undergo Warburg‐like metabolic transitions during renal disease. Our single‐cell analysis further reveals that this metabolic reprogramming is highly heterogeneous across cellular subpopulations [20–22]. Yet, our single‐cell resolution uncovers that this metabolic reprogramming is not homogeneous but rather it exerts an heterogeneous impact among cellular subpopulations, hinting that the metabolic state could be a major sprouting determinant of cell fate decisions [23]. Metabolic transition from OXPHOS to glycolysis as detected in our study may be a process with two faces rather than being purely maladaptive. In these acute injuring stages, glycolytic metabolism could act as a “first line of defense” by decreasing oxidative stress, sustaining ATP generation under hypoxic condition and promoting cell survival in the event of an impaired mitochondrial function. However, chronic glycolytic reprogramming during disease progression is deadaptive and leads to cellular dysfunction, undifferentiation, and activation of profibrotic pathways. This timing is important for explaining how metabolic adaptation moves from a good to a bad situation, and it may be relevant in terms of therapeutic interventions aimed at preserving the acute protection while preventing long‐term maladaptation. The differential expression of metabolic enzymes such as lactate dehydrogenase isoforms (LDHA and LDHB) has been shown to be the molecular basis for divergent TKs strategies in disease progression, consistent with reciprocal changes of specific genes in glycolytic pathway [23, 24]. Cells with high post‐GC LDHB expression retained oxidative respiratory capacity and may be more resistant or less severely injured, compared to the cells with overexpressed LDHA, which switched from mitochondrial respiration to glycolysis[c] in order to compensate for rupture of functional mitochondria (hypoxia) [25]. The presence of cells with high mitochondrial transcript fraction and low TCA cycle enzyme expression points to induced mitochondrial stress and/or “dysfunction,” possibly occurring alongside a transitional phase before complete metabolic breakdown or death. The physiological significance of metabolic reprogramming in CKD is not limited to energy generation. Glycolysis as a metabolic process is related to the activation of profibrotic pathways, modification of the cellular redox state, and alteration in epigenetic regulation by metabolite induced modifications [20, 21]. Thus, the metabolic heterogeneity we detected is likely to have implications for the tubular response to injury as well, with certain populations being predisposed for profibrotic transformation, while other may retain regenerative potential. Expression patterns of segment‐specific markers such as SLC34A1, SLC5A2, LRP2, CUBN, and ALDOB and GATM described here demonstrate the loss of tubular specialization with increasing damage and diseased state. The finding of reduced SLC34A1 expression is in keeping with previous proteomic [26] and immunohistochemistry studies in human CKD biopsies demonstrating loss of this sodium phosphate cotransporter within diseased proximal tubules. Consistent with the above result, the upregulation of ANXA1 we detected is consistent with immunohistochemical findings demonstrating increased annexin A1 protein expression in tubular epithelial cells of fibrotic regions in CKD kidneys, implicating this inflammation‐regulatory mediator in CKD progression. This observation has important functional implications because the aberrant expression of transporters and enzymes that are key for reabsorptive processes leads directly to the various clinical features of CKD, such as alterations in plasma concentrations of electrolytes, proteinuria, and changes in amino acid metabolism. This preservation of markers in some cells and complete loss in others is consistent with the notion that dedifferentiation does not have to occur under every circumstance and points toward potential therapeutic strategies targeting sequestration of the differentiated phenotype. The signals that precipitate the loss of segmental identity probably include a web of crosstalk between metabolic stress, inflammation, and epigenetic reprogramming. Through our trajectory analysis, we uncovered the regulatory significance of distinct and conserved regulation genes such as MALAT1 and ANXA1, implying that these molecules may have conserved functions in maintaining or disrupting differentiated states. As a highly conserved long noncoding RNA, MALAT1 may even serve as fundamental “housekeeping” or structural functions across cell states [27, 28], while the identification of the variable importance in ANXA1 suggests their specific role within inflammation‐driven cellular transitions [26, 29]. An analysis of the ligand–receptor interactions illuminated complex intercellular communication networks likely to be involved in coordinating injury responses among diverse cell types. Identification of signaling pathways that differ among cell types and disease states may offer therapeutic strategies to regulate disease. Yet, the intricate network of these pathways also demonstrates how difficult it would be to identify ideal points of intervention as blockade of one pathway can result in compensatory activation through another signaling cascade. The enrichment of injury‐associated and profibrotic signatures in certain proximal tubule clusters implies that discrete cellular subpopulations may be the principal drivers of fibrogenesis, either by directly transdifferentiating into myofibroblast‐like cells or through paracrine signaling to drive resident fibroblasts. In future works, integration of spatial transcriptomics with single‐cell analysis will be indispensable to dissect these cellular crosstalks at the level of anatomic arrangements and identify where in the anatomy pathological processes are launched or amplified. Despite the complexity of the overlap among cellular states, the fact that machine learning classifiers achieve only moderate performance (ROC AUC = 0.673) shows that automated cell type identification in kidney disease is feasible. Although moderate AUCs are consistent with the difficulty of discriminating between similar cellular states in a continuous landscape of differentiation, the common level performance for different training fractions indicates the ability of these models to represent biologically relevant structure. This classifier could be used on other studies for fast cell type annotation, and in the future to diagnostic tools if coupled to affordable technologies such as targeted RNA profiling. The plateauing learning curves suggest that further improvements in classification are more likely to result from improved feature selection or inclusion of additional data modalities, rather than simply increasing sample size. Inclusivity of measurements such as protein expression, chromatin accessibility, or spatial positioning could contribute to orthogonal features that would discriminate less well‐separated cellular states.

## 5. Limitations

There were some limitations of this study that should be considered. First, the cross‐sectional design of our analysis captures cellular states at a moment in time and, although we cannot prove that these changes are causative or that they develop over time during disease progression. Lack of RNA velocity impedes us from conclusively verifying the directionality and dynamics of the inferred cellular transitions. In the future, the application of RNA velocity approaches (e.g., scVelo) could also be incorporated in order to capture kinetic evidences of states transitions by spliced versus unspliced mRNA ratios, which would support trajectory‐derived conclusions. Longitudinal studies of individuals or animal models over time would enhance causal inference for cellular transitions [30]. First, our metabolic states are assigned not by the analytical scores of specific pathways but rather majorly through observing expression levels of metabolism‐related genes. Although we noticed stark metabolic dichotomies, a more thorough quantification of individual metabolic activities (both OXPHOS and fatty acid oxidation as well as glycolysis) could be achieved by gene set variation analysis (GSVA) or single‐sample gene set enrichment analysis (ssGSEA) [31]. Selective investigation of rate‐controlling enzymes (such as CPT1A for fatty acid oxidation) would further clarify the functional importance of metabolic reprogramming and defective FAO in tubular dysfunction in CKD. Third, the dissociation required for scRNA‐seq may introduce stress‐related transcriptional artifacts and result in loss of spatial context. Lack of spatial validation makes it difficult to definitively determine how the discovered cell states contribute to local fibrotic milieu. Because CKD is driven by focal and interstitial fibrosis, the spatial correlation between injured tubular epithelial cells and the profibrotic niche is important. Immunofluorescence or multiplex immunohistochemistry analyses to confirm the spatial distribution of defined tubular epithelial cell subclusters (e.g., injury‐associated cells, or acknowledge differentiating cells) would be necessary to underpin the proposed hypothesis that these specific cellular states contribute toward local fibrotic remodeling. Integrated spatial transcriptomics approaches can provide important context on the anatomical organization of cellular heterogeneity and cell–cell interactions in situ.

## 6. Conclusion

This study provides a comprehensive single‐cell transcriptomic atlas revealing extensive heterogeneity within tubular epithelial cells during CKD progression. We demonstrate that tubular cells exist in multiple functional and metabolic states characterized by differential expression of segment‐specific markers and metabolic enzymes, with clear metabolic dichotomy between OXPHOS‐dominant and glycolytic populations.

## Author Contributions

Xinhui Mao conducted primary research, performed data analysis, and drafted the manuscript; Minggang Wei contributed to the experimental design, data interpretation, and critical revision of the manuscript; Yilin Li provided clinical insights, contributed to data collection, and assisted with statistical analysis; Ping Xia supervised the research, designed the study, and takes academic responsibility for the work as corresponding author.

## Funding

This work was supported by the 2024 Suzhou Applied Basic Research Science and Technology Innovation Project (Grant No. SYWD2024278) and the 2024 Research Projects of the Jiangsu Association of Traditional Chinese Medicine (Grant No. CYTF2024042).

## Ethics Statement

This study utilized publicly available data from the Gene Expression Omnibus (GEO) database. As the analysis involved only deidentified data from public repositories, ethics approval was not required according to institutional guidelines.

## Conflicts of Interest

The authors declare no conflicts of interest.

## Data Availability

The data that support the findings of this study are available from the corresponding author upon reasonable request.
